# *In Ovo* Injection of CHIR-99021 Promotes Feather Follicle Development *via* Modulating the Wnt Signaling Pathway and Transcriptome in Goose Embryos (*Anser cygnoides*)

**DOI:** 10.3389/fphys.2022.858274

**Published:** 2022-05-20

**Authors:** Ziqiang Feng, Haizhou Gong, Jinhong Fu, Xiaohui Xu, Yupu Song, Xiaomin Yan, Ichraf Mabrouk, Yuxuan Zhou, Yudong Wang, Xianou Fu, Yujian Sui, Tuoya Liu, Chuanghang Li, Zebei Liu, Xu Tian, Le Sun, Keying Guo, Yongfeng Sun, Jingtao Hu

**Affiliations:** ^1^ College of Animal Science and Technology, Jilin Agricultural University, Changchun, China; ^2^ Key Laboratory of Animal Production, Product Quality and Security (Jilin Agricultural University), Ministry of Education, Changchun, China; ^3^ College of Veterinary Medicine, Jilin Agricultural University, Changchun, China

**Keywords:** CHIR-99021, feather follicle, in ovo injection, goose, wnt signaling pathway, transcriptome

## Abstract

Feather performs important physiological functions in birds, and it is also one of the economic productions in goose farming. Understanding and modulating feather follicle development during embryogenesis are essential for bird biology and the poultry industry. CHIR-99021 is a potent Wnt/β-catenin signaling pathway activator associated with feather follicle development. In this study, goose embryos (*Anser cygnoides*) received an in ovo injection of CHIR-9902, which was conducted at the beginning of feather follicle development (E9). The results showed that feather growth and feather follicle development were promoted. The Wnt signaling pathway was activated by the inhibition of GSK-3β. Transcriptomic analyses showed that the transcription changes were related to translation, metabolism, energy transport, and stress in dorsal tissue of embryos that received CHIR-99021, which might be to adapt and coordinate the promoting effects of CHIR-99021 on feather follicle development. This study suggests that *in ovo* injection of CHIR-99021 is a potential strategy to improve feather follicle development and feather-related traits for goose farming and provides profiling of the Wnt signaling pathway and transcriptome in dorsal tissue of goose embryos for further understanding of feather follicle development.

## Graphical Abstract

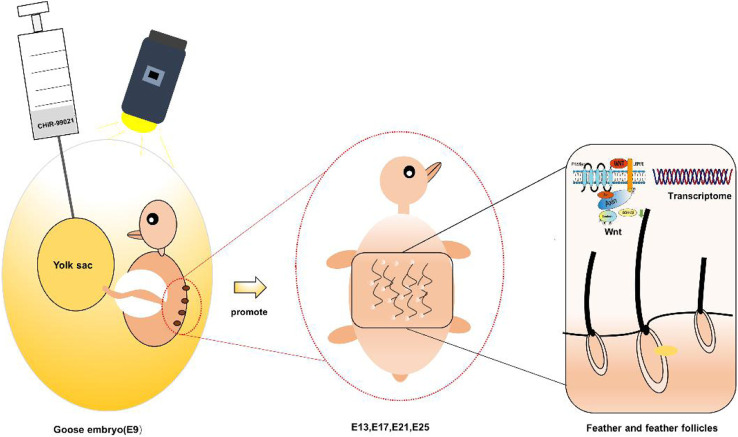

## Introduction

Feather supports functions of endothermy, communication, and flight in birds, and it is also one of the economic productions in goose farming (Chen, et al., 2015). Understanding and modulating feather growth and feather follicle development are of significance to the fields of bird biology and the poultry industry. Feather follicles, as a complex micro-organ, govern feather growth and development during the embryonic period through signal communication between the epidermis and dermis. During the developmental process of feather formation, transduction molecules, transcription factors, and other genetic factors synergistically and/or independently influence the dynamic morphogenesis of skin and feather follicles (Inamatsu, et al., 2006; Jiang, et al., 2011). Studies have confirmed that the Wnt, TGF-β, MAPK, and BMP signaling pathways are candidate morphogens associated with feather follicle development during embryogenesis (Chen, et al., 2020a; Ji, et al., 2021; Zheng, et al., 2020). Particularly, the Wnt is the initial signal for feather follicle development and participates in each stage of feather follicle morphogenesis, such as placode formation, dermal papillae function, feather follicle cycle, and proliferation and differentiation of feather follicle stem cells (Andl, et al., 2002; García de Herreros and Duñach, 2019; Huelsken, et al., 2001; Kaushal, et al., 2015; Nusslein-Volhard and Wieschaus, 1980; Telerman, et al., 2017; Tsai, et al., 2014; Wong, et al., 1994; Wu, et al., 2020).

Glycogen synthase kinase 3β (GSK-3β) is a potential target for modulating development. Previous studies have shown that GSK-3β is the main regulator participating in some cellular pathways consisting of insulin signaling and glycogen synthesis, neurotrophic factor signaling, neurotransmitter signaling, and microtubule dynamics (Beaulieu, et al., 2007; Forde and Dale, 2007; Phiel, et al., 2003). Moreover, GSK-3β plays important roles in metabolism, transcription, development, cell survival, and neuronal function (Kempson, et al., 2017; Naujok, et al., 2014; Wu, et al., 2015), as well as in the Wnt signaling pathway (Rayasam, et al., 2009).

Small molecules with membrane permeability have been applied in the maintenance and differentiation of stem cells (Wu, et al., 2015). CHIR-99021 is a potent and selective GSK-3α/β inhibitor and the Wnt/β-catenin signaling pathway activator (Naujok, et al., 2014; Ring, et al., 2003; Ye, et al., 2012), stabilizes and influences β-catenin in the Wnt/β-catenin pathway, regulates several other pluripotency-related pathways (such as TGF-β, Notch, MAPK, and BMP), and participates in embryonic development and differentiation (Wu, et al., 2013). Advantageously, CHIR-99021 changes epigenetic expression regulation genes and long intergenic non-coding RNA (Wu, et al., 2013), while it does not produce significant concomitant toxicity (Naujok, et al., 2014; Wu, et al., 2013). Moreover, *in ovo* injection is an efficient way to intervene in embryonic development (Chen, et al., 2020b; Slawinska, et al., 2016; Xie, et al., 2020b). Therefore, to understand and regulate the development of feather follicles in geese, this study adopted *in ovo* injection of CHIR-99021 to goose embryos at the beginning of feather follicle development and assessed the effects of CHIR-99021 on feather indices, feather follicle development, the Wnt signals, and dorsal skin transcriptome in goose embryos.

## Materials and Methods

### Experimental Animals

This experiment was approved by the Animal Health Care Committee of Animal Science and Technology College of Jilin Agricultural University (Approval No. GR (J) 18–003). Breeder eggs of Zi goose (*Anser cygnoides*) were obtained from the geese breeding research center, Jilin Agricultural University (Changchun, China), and incubated in an incubator in laboratory according to the routine procedure.

### Experimental Design and Treatments

Candling procedure was conducted on day 6 of incubation to identify and remove the unfertilized eggs and the dead embryos. In the pre-experiment, 60 fertilized eggs were used to observe the feather follicle morphogenesis (from E9 to E25). Then, 400 fertilized eggs were divided into five groups of 80 eggs each *in ovo* injection experiments. The different doses of CHIR-99021 solutions were prepared and preheated to 37.9°C before injecting (E9). Then, the CHIR-99021 solutions were injected into the egg yolk sac. The group names were abbreviated according to the dose they received, consisting of 1) non-injection group (BLANK group); 2) injection of 100 μl of 0.9% normal saline (NS)/egg (CK group); 3) injection of 100 μl of 0.9% NS/egg containing 1,000 ng CHIR-99021/egg (1,000 ng group); 4) injection of 100 μl of 0.9% NS/egg containing 2,000 ng CHIR-99021/egg (2000 ng group); 5) injection of 100 μl of 0.9% NS/egg containing 5,000 ng CHIR-99021/egg (5,000 ng group).

### Preparation of CHIR-99021 Solutions and the *In Ovo* Injection Procedure

**FIGURE 1:**
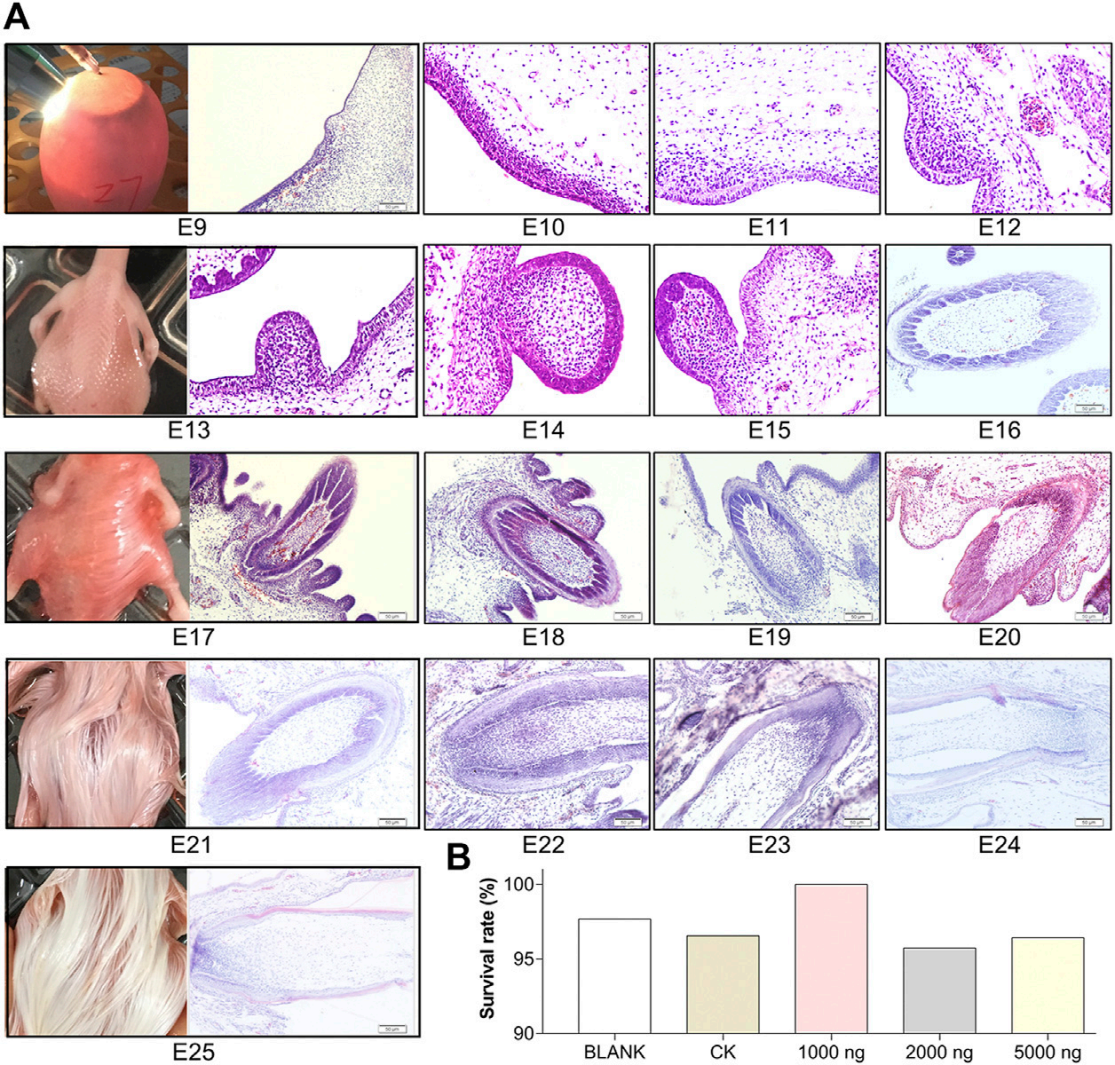
Feather follicle morphogenesis determines the time nodes for goose embryos in ovo injection and sampling. **(A)** Representative images of feather follicle morphology during E9 to E25 as indicated by H&E, operation of *in ovo* injection at E9, and dorsal feather phenotypes at E13, E17, E21, and E25. Bar = 50 μm. **(B)** Survival rate of each group (data from 50 fertilized eggs of each group).

CHIR-99021 (CHIR-99021 monohydrochloride, MedChemExpress, Shanghai, China) was dissolved in 0.9% NS. The center of the large end of each egg was located under the egg candler and an alcohol swab was used to disinfect the target injection site, then a small hole was drilled at the injection site. As shown in Figure 1A, the volume of 100 μl of the CHIR-99021 solution was rapidly injected into the egg yolk sac using a 0.7 × 30 mm needle. After paraffin sealing, the eggs were returned to the incubator immediately. The non-injection group eggs also stayed outside the incubator environment for 15 min for standardization.

### Sampling and Measurements of Feather Length and Feather Width

The dorsal skin tissue samples (about 1.5 cm^2^, the cross region of the dorsal midline) were obtained during 0–1 h at E13, E17, E21, and E25. Survivability was the percentage of surviving embryos in each group of the total number of fertilized eggs. A part of the skin tissue was frozen in liquid nitrogen and stored at −80°C for western blotting and RNA sequencing (RNA-seq) analyses. The samples were mixed from three embryos to ensure sufficient material for molecular biology assays. Other part of the skin tissue was fixed in 4% paraformaldehyde for further histological observations.

The feathers were obtained at E17, E21, and E25. The sampling region was the cross region of the dorsal midline. The collected feathers were cleaned with PBS. A part of the feathers was used for feather length measurement using a vernier caliper (precision 0.01 mm). Other part of feathers was stuck on slides for feather width measurement. The width of feather center was measured using CU-6 fiber fineness meter (Olympus, Tokyo, Japan).

### Morphological Observations and Measurements of Feather Follicle Diameter and Feather Follicle Density

Feather follicle morphological observations were conducted using hematoxylin and eosin (H&E) staining. The fixed tissues were dehydrated in different concentrations of ethanol (50,75, 85, 90, 95, and 100%) and were soaked twice in xylene for clearing. The tissues were embedded in paraffin according to conventional methods.

Paraffin-embedded 5 μm sections of dorsal skin tissues from E9 to E25 were prepared. The sections were stained using H&E according to standard methods and were photographed using a microscope (Olympus, Tokyo, Japan).

The distribution of feather follicles was observed and photographed from the longitudinal skin sections to measure feather follicle diameter using microscope software (cellSens v.3.1, Olympus, Tokyo, Japan). The distance of the outermost layer is the maximum in feather follicle and was measured to assess the feather follicle diameter (red arrows in Figure 3A). A total of 10 regions of each transverse skin section were photographed to calculate the number of feather follicles, which was used to calculate the feather follicle density.

### Western Blotting Analysis

The total protein samples for electrophoresis were extracted using RIPA buffer (Beyotime Biotechnology, Shanghai, China), and the total protein concentration was determined using a BCA protein concentration detection kit (Beyotime Biotechnology). For each sample, the volume containing 10 μg total proteins was pipetted onto a 10% SDS-PAGE. The proteins were transferred to the surface of the PVDF membrane. The membranes were blocked with 5% skim milk and then incubated with primary antibodies at 4°C overnight. The antibodies identifiers and dilutions were as follows: rabbit anti-β-catenin antibody (Cat# bs-23663R, bioss, Beijing, China), rabbit anti-GSK-3 β antibody (Cat# bs-0023M, bioss), 1:2,000,1:2,000; rabbit anti-c-Myc antibody (Cat# bs-4963R, bioss), 1:2,000; rabbit anti-TCF4 antibody (Cat# bs-1280R, bioss), 1:2000; rabbit anti-LEF1 antibody (Cat# bs-1843R, bioss), 1:1,500; rabbit anti-FZD4 antibody (Cat# bs-13217R, bioss), 1:2,000; ACTB rabbit monoclonal antibody (Cat# AC026, Abclonal, Wuhan, China), 1:50,000. At the end, the membranes were incubated with a goat anti-rabbit IgG (H + L)/HRP antibody (Biosynthesis Biotechnology, Beijing, China, 1:50,000 dilution) for 1 h at RT. Goose is not a common experimental animal, which causes a limitation of commercial antibodies. The antibodies reacting with chicken were used in the study. A prior experiment verified that these antibodies can specifically react with goose. The molecular weights between some proteins are nearby, stripping, and thereby was used to avoid errors cross gels. A stripping buffer (Solarbio, Beijing, China) was used for 12 min to strip the antibodies bound on the membranes, then blocking, and antibody incubating were conducted as described above. Finally, the membranes were visualized with an ECL Test Kit (Millipore, Darmstadt, Germany) under a Bio-Rad imaging system (Bio-Rad, Hercules, CA, United States). The chemiluminescence of each protein blotting was quantified using the ImageJ software (NIH, Bethesda, MD, United States). The protein levels were normalized by β-actin as an internal control.

### Immunohistochemical Observations

The slides with 5-μm thick paraffin-embedded dorsal skin tissues sections were baked at 65°C for 3 h, dewaxed twice in xylene, and hydrated in descending grades of alcohol. Then, the slides succumbed to antigen retrieval by boiling in sodium citrate buffer (pH 6.0) for 20 min after deparaffinization and hydration and were rinsed with PBS for 5 min of 3 times at the end of each step. Following the steps of deparaffinization, rehydration, and antigen retrieval, the sections were blocked with 3% hydrogen peroxide at RT for 20 min and with 3% normal goat serum at RT for 20 min, and were incubated overnight at 4°C with 50 μl of rabbit anti-β-catenin antibody (Cat# bs-23663R, bioss, Beijing, China) at 1:100 diluent. Then, the sections were incubated, respectively, with a secondary antibody linked with biotin and the HRP-linked streptomycin according to UltraSensitive™ SP Rabbit IHC Kit (MXB, Fuzhou, Fujian, China) instructions. DAB (MXB, Fuzhou, Fujian, China) and hematoxylin were, respectively, used for chromogen/substrate and nuclear staining. Finally, the slides were observed and photographed using a microscope system (Olympus, Tokyo, Japan) after coverslipping.

### RNA Extraction and RNA-Seq

The dorsal skin tissue samples from CK and the 5,000 ng group at E13 and E25 were used for RNA-seq. Total RNA was extracted using a Trizol reagent kit (Invitrogen, Carlsbad, CA, United States) according to the manufacturer’s protocol. The harvested RNA was quantified using a nanodrop spectrophotometer (BIO-DL, Shanghai, China). The RNA quality was assessed on an Agilent 2,100 Bioanalyzer (Agilent Technologies, Palo Alto, CA, United States) and agarose gel electrophoresis. The RNA integrity numbers (≥7.3) and concentrations (≥211 ng/μl) met the requirements of library construction (Supplementary Table S1). After total RNA extraction, eukaryotic mRNA was enriched by oligo (dT) beads, while prokaryotic mRNA was enriched by removing rRNA using Ribo-Zero™ Magnetic Kit (Epicentre Biotechnologies, Madison, WI, United States). Then, the enriched mRNA was fragmented into short fragments using fragmentation buffer and reverse transcribed into cDNA with random primers. Second-strand cDNA was synthesized by DNA polymerase I, RNase H, dNTP, and buffer. Then, the cDNA fragments were purified with a QiaQuick PCR extraction kit (QIAGEN, Dusseldorf, Germany), and were end-repaired, poly(A) added and ligated to Illumina sequencing adapters. The size of ligation products was selected using agarose gel electrophoresis. The purified amplicons were sequenced using Illumina HiSeq™ 4,000 platform by Gene Denovo Biotechnology Co. (Guangzhou, China).

### *De Novo* Transcriptome Analyses

The qualities of the raw reads were checked using fastp software (version 0.18.0) (Chen, et al., 2018). The reads containing adapters, reads containing more than 10% unknown nucleotides (N), and low-quality reads containing more than 50% of low-quality (Q-value ≤ 10) bases were removed. Then, a total of 538502706 raw reads and 536003454 clean reads were obtained (clean reads rates ≥99.49%). The bases Q20 (≥98.28%), Q30 (≥95.13%), and GC content (53.42–56.98%) in clean reads were within reasonable ranges (Supplementary Table S2). Next, the clean reads were mapped to RNA to identify and remove residual rRNA reads. Then, the rRNA removed high-quality clean reads and was further used for assembly and gene abundance calculations. The high-quality clean reads were mapped to the reference transcriptome using Bowtie2 software by default parameters (version 2.2.8) (Langmead and Salzberg, 2012). The clean reads assembled 91,236 unigenes from 93525236 bases by using Trinity rna-seq software (version 2.13.2) (Grabherr, et al., 2011). To get the protein function annotation information of the unigenes with the highest sequence similarity (Ashburner, et al., 2000; Conesa, et al., 2005), the unigenes were annotated to Nr, SwissProt, KEGG, and COK/KOG databases by using Blastx (NCBI, Bethesda, MD, United States). The number of homologous sequences between species, and these unigenes’ function annotations are shown in Supplementary Figure S1.

The gene abundance was calculated and normalized by RPKM (reads per kb per million reads) using RESM software (version: 1.3.3) (Mortazavi, et al., 2008). To identify differentially expressed genes between groups, the edgeR package was used. We identified genes with a fold change ≥2 and a *p* < 0.05 in comparison as differentially expressed genes (DEGs). The DEGs were then subjected to enrichment analysis of GO terms and KEGG pathways (Kanehisa, et al., 2008). The GO terms and KEGG pathways with *p* < 0.05 were defined as significantly enriched terms and pathways.

The differently expressed transcription factors were classified and calculated according to their families. The shared and unique differently expressed transcription factors between E13 and E25 were ranked by -log_10_ (*p*-value) and presented as a heatmap. The PPI network of the differently expressed transcription factors was constructed using STRING (https://www.string-db.org) with confidence >0.9.

### Validation of *De Novo* Transcriptome by q-PCR

The number of 16 DEGs screened by *de novo* transcriptome was randomly selected to conduct q-PCR validation. The primers are listed in Supplementary Table S3. The cDNA was synthesized for q-PCR using a PrimeScript RT Reagent Kit with gDNA Eraser (Morey Biosciences, Shanghai, China). q-PCR was performed with SYBR Green Reagent (Morey Biosciences, Shanghai, China) in a CFX Connect Real-Time PCR Detection System (Bio-Rad, Hercules, CA, United States). The total volume of q-PCR reactions was 20 μl comprising 2 μl of cDNA, 0.6 μl of both forward, and reverse primers, 6.8 μl of RNase-free H_2_O, and 10 μl SYBR Green Reagent. The amplification conditions were as follows: pre-denaturation at 95°C for 5 min, then 40 cycles of amplification (95°C for 15 s and 59°C for 60 s). Expression was quantified using the 2^–ΔΔCt^ method. The β-actin gene was chosen as the control gene to normalize the mRNA expression levels. The q-PCR results supported the reliability of *de novo* transcriptome in this study (Supplementary Figure S2).

### Statistical Analyses and Visualization

The statistical tests were performed with SPSS 23.0 software (IBM, Armonk, NY, United States). The data were visualized using the GraphPad Prism 8 software (GraphPad, San Diego, CA, United States). The statistical significance was determined using one-way ANOVA by Duncan’s multiple range test. The significant difference in the data was considered as *p* < 0.05. The results were expressed as mean ± SEM in the figures.

## Results

### Feather Follicle Morphogenesis Determines the Time Nodes for Goose Embryos *In Ovo* Injection and Sampling

To determine the time nodes for *in ovo* injection and sampling, feather follicle morphology was observed during E9 to E25. As shown in Figure 1A, the dermal cell condensation appeared from E9 and that was considered the outset of feather follicle development. The short bud structure started to format at E13. By E17, the stage from the cavity to follicle started and the long bud structure started sinking into the epidermis. Feather follicles have sunk into the epidermis and developed basic structures by E21. At E25, the feather follicles were completely developed, and at this period, the muscles, nerves, glands, and blood vessels connected with mature feather follicles gradually become abundant in the dermis. Therefore, E9 was chosen as the time node for *in ovo* injection; E13, E17, E21, and E25 were chosen as the time nodes for sampling. The survival rates in the embryos that received CHIR-99021 injection were above 95% (Figure 1B), which suggested that CHIR-99021 and the doses were in a safe range for embryos in this study.

### *In Ovo* Injection of CHIR-99021 in Goose Embryos Promotes Feather Growth and Feather Follicle Development

**FIGURE 2:**
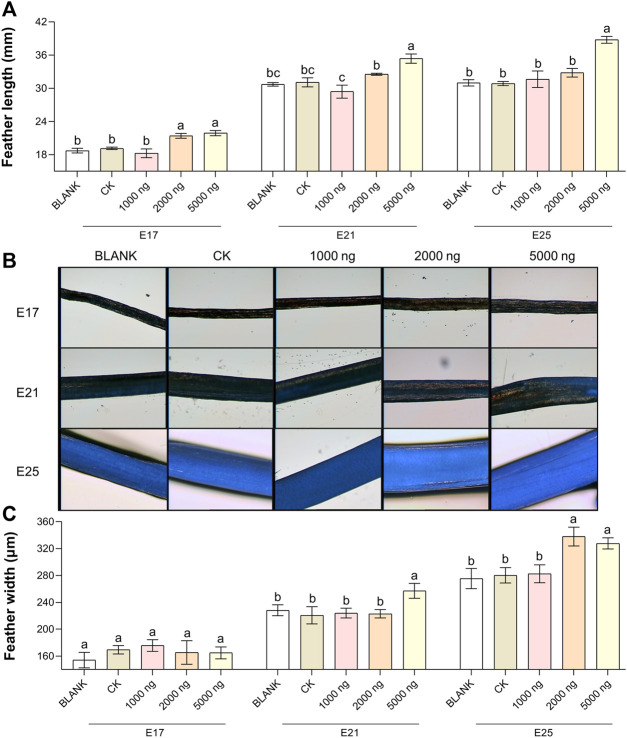
In ovo injection of CHIR-99021 in goose embryos promotes feather growth. **(A)** Feather length of each group at E17, E21, and E25. **(B)** Representative images of feather width. **(C)** Feather width of each group at E17, E21, and E25. All data are shown as mean ± SEM; no common superscript letter within the same embryonic day means statistical difference (*p* < 0.05) by one-way ANOVA with Duncan’s multiple-range test. n = 6 embryos.

The feather length results (Figure 2A) and feather width results (Figures 2B,C) suggested that *in ovo* injection of CHIR-99021 had the ability to promote feather growth. Compared with the BLANK and CK groups, *in ovo* injection of 5,000 ng CHIR-99021 significantly (*p* < 0.05) promoted the feather length at E17, E21, and E25, while *in ovo* injection of 2,000 ng CHIR-99021 also had significantly (*p* < 0.05) promoted effect on feather length at E17. Moreover, feather width in the 5,000 ng CHIR-99021 group at E21 and E25 and in the 2,000 ng CHIR-99021 group at E25 were significantly (*p* < 0.05) increased.

**FIGURE 3:**
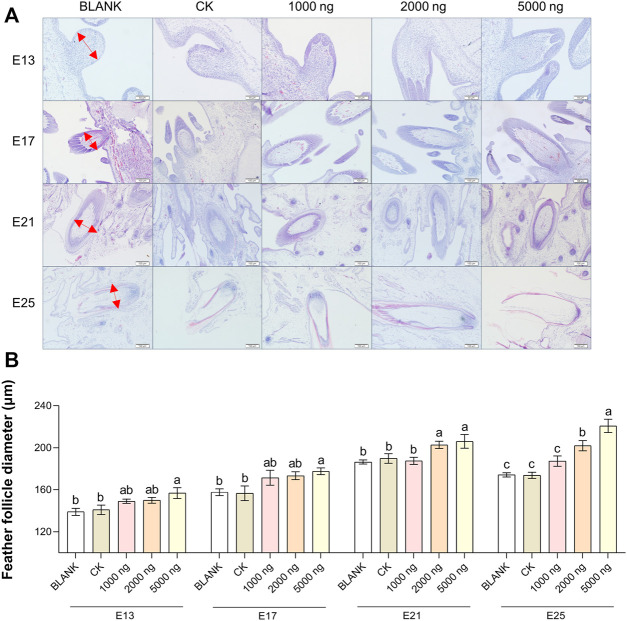
In ovo injection of CHIR-99021 in goose embryos promotes feather follicle diameter. **(A)** Representative images of feather follicle diameter as indicated by H&E. The red arrows signal measured distance. Bar = 50 μm at E13 and E17, Bar = 100 μm at E21 and E25. **(B)** Feather follicle diameter of each group at E13, E17, E21, and E25. All data are shown as mean ± SEM; no common superscript letter within the same embryonic day means statistical difference (*p* < 0.05) by one-way ANOVA with Duncan’s multiple-range test. n = 6 embryos.

**FIGURE 4:**
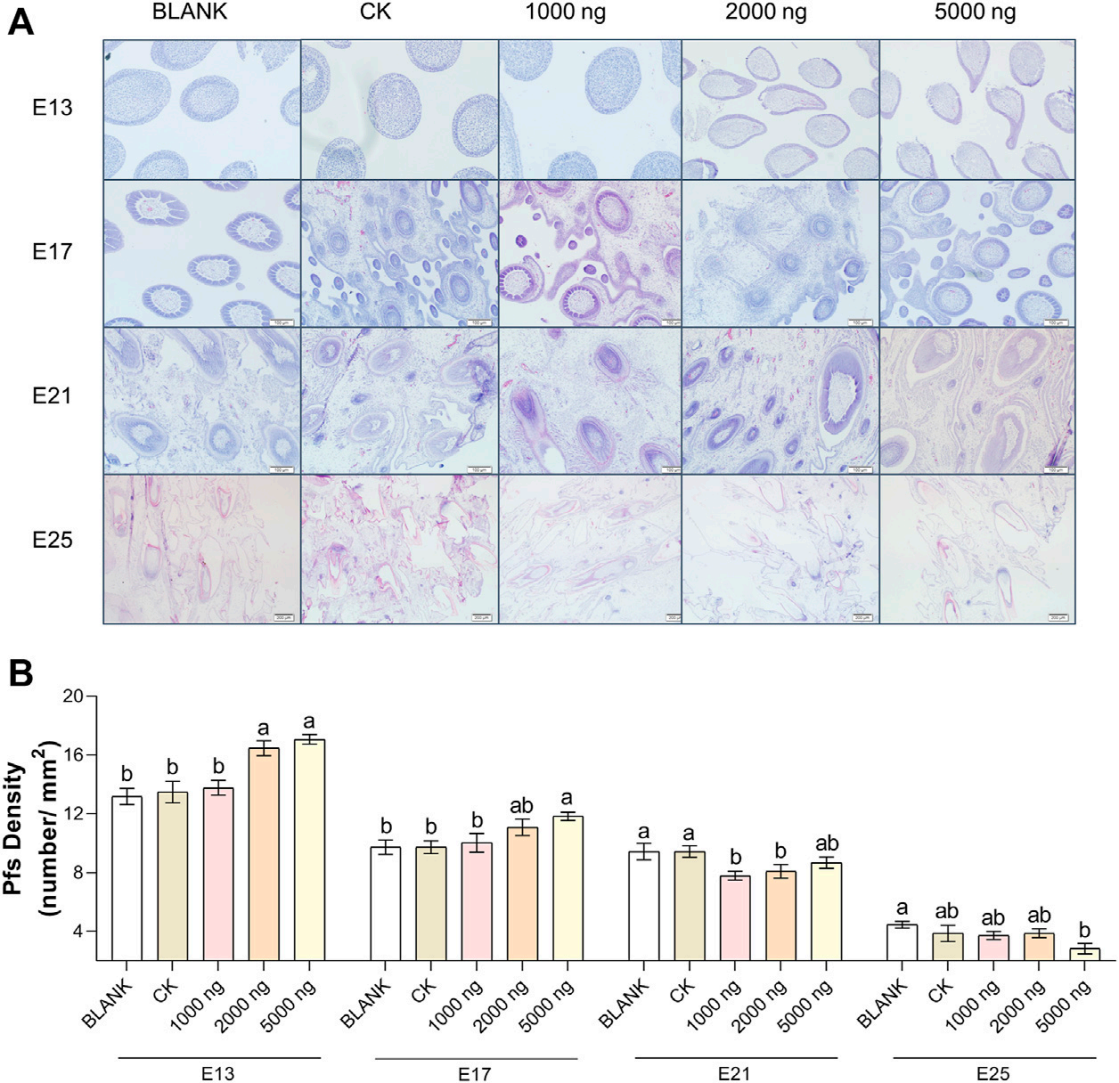
In ovo injection of CHIR-99021 in goose embryos promotes feather follicle density. **(A)** Representative images of feather follicle density as indicated by H&E. Bar = 50 μm at E13 and E17, Bar = 100 μm at E21 and E25. **(B)** Primary feather follicle density of each group at E13, E17, E21, and E25. All data are shown as mean ± SEM and no common superscript letter within the same embryonic day means statistical difference (*p* < 0.05) by one-way ANOVA with Duncan’s multiple-range test. n = 6 embryos.

The feather follicle diameter (Figure 3) and feather follicle density (Figure 4) results suggested that CHIR-99021 also promoted feather follicle development. Morphological observations were conducted to investigate feather follicle development during morphogenesis between the groups (Figures 3A, 4A). The results (Figure 3B) showed that feather follicle diameter in the 5,000 ng CHIR-99021 group was significantly (*p* < 0.05) wider than that in the BLANK and CK groups at E13, E17, E21, and E25. The feather follicle diameter in the 2,000 ng group was significantly (*p* < 0.05) promoted at E17, E21, and E25, and that in the 1,000 ng group was significantly (*p* < 0.05) increased at E25. The results of primary feather follicles (Pfs) density (Figure 4B) showed that injection of 5,000 ng CHIR-99021 significantly (*p* < 0.05) increased Pfs density at E13 and E17, and injection of 2,000 ng CHIR-99021 significantly (*p* < 0.05) increased Pfs density at E13. However, the Pfs densities in the 1,000 ng group at E21 and in the 5,000 ng group at E25 were significantly (*p* < 0.05) decreased and the Pfs densities in other injected groups were at a common level (*p* > 0.05) with the BLANK and CK groups. These were possibly associated with their wider feather follicle diameter leading to a decreased number of feather follicles in the same field of view.

### *In Ovo* Injection of CHIR-99021 in Goose Embryos Activates the Wnt Signaling Pathway via Inhibiting GSK-3β

**FIGURE 5:**
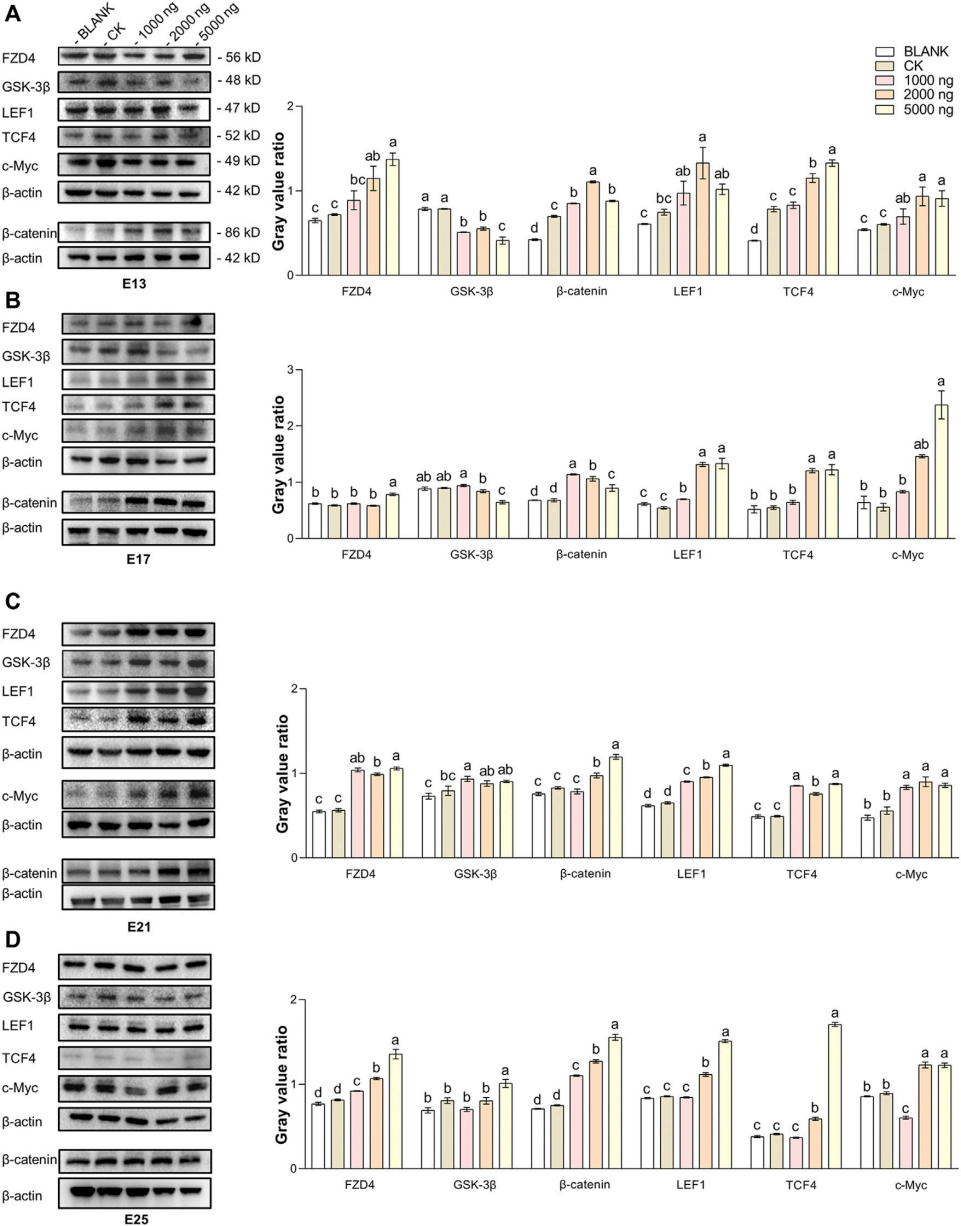
In ovo injection of CHIR-99021 in goose embryos activates the Wnt signaling pathway by inhibiting GSK-3β. The expression of FZD4, β-catenin, LEF1, TCF4, and c-Myc in dorsal tissues of each group at E13 **(A)**, E17 **(B)**, E21 **(C),** and E25 **(D)**, as detected by western blotting. All data are shown as mean ± SEM, no common superscript letter within the same embryonic day means statistical difference (*p* < 0.05) by one-way ANOVA with Duncan’s multiple-range test. n = 6 embryos.

The expression of hub molecules in the Wnt/β-catenin signaling pathway in the dorsal tissues was investigated using western blotting between groups (Figure 5). At E13 (Figure 5A), injection of CHIR-99021 significantly (*p* < 0.05) inhibited the expression of GSK-3β in the embryonic dorsal tissues. A dose of 5,000 ng had more significantly inhibited the effect compared with the doses of 1,000 ng and 2,000 ng. In addition, CHIR-99021 had the ability to upregulate the expression of FZD4, β-catenin, the transcription factors LEF1 and TCF4, and the target c-Myc in the Wnt signaling pathway. Moreover, similar regulations of these Wnt molecules were found at E17 and E21 (Figures 5B,C). However, by E25 (Figure 5D), the expression of GSK-3β in the 1,000 ng and 2,000 ng groups was at a common level (*p* > 0.05) compared with the BLANK and CK groups and the level of GSK-3β in the 5,000 ng group were significantly (*p* < 0.05) increased. This might associate with complete feather follicle morphogenesis and injection timing. The levels of FZD4, β-catenin, LEF1, TCF4, and c-Myc maintained an upregulated trend in the groups that received CHIR-99021 injection. These results suggested that, at the level of protein, *in ovo* CHIR-99021 injection promoted feather growth and feather follicle development by inhibiting GSK-3β and upregulating FZD4, β-catenin, LEF1, TCF4, and c-Myc. The raw western blotting images are attached in Supplementary Material S1.

**FIGURE 6:**
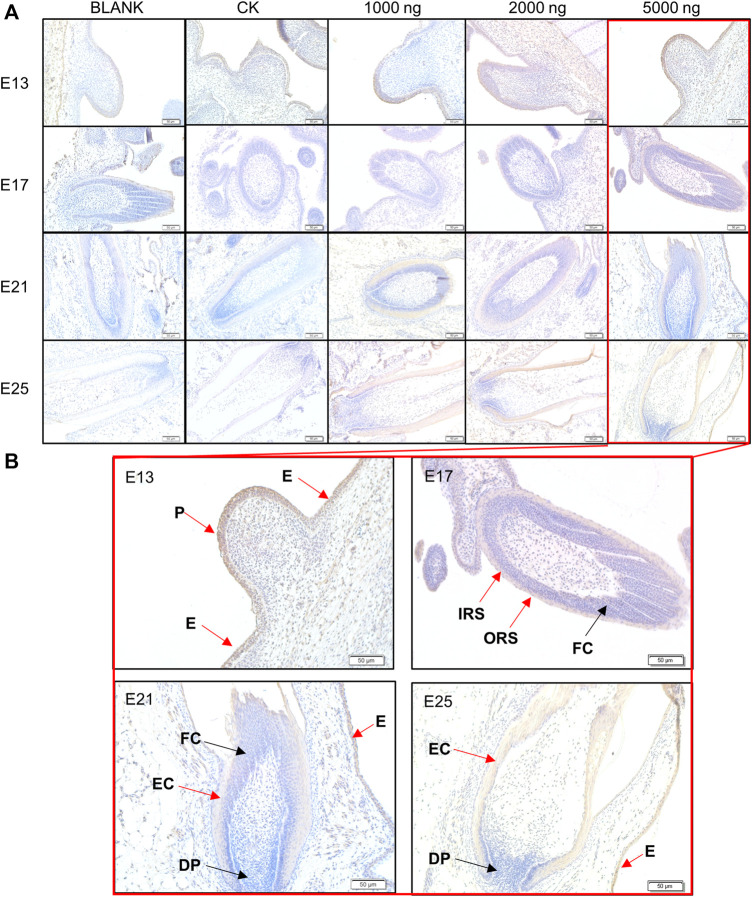
**(A)** Representative images of distribution of beta-catenin in feather follicles as detected by IHC-P. **(B)** The main positive expression area of β-catenin. Black arrows: negative express, red arrows: positive express. P, Placode; DP, Dermal papilla; E, Epidermis; EC, Epidermal collar; FC, Feather crest; IRS, Inner root sheath; ORS, Outer root sheath. Bar = 50 μm.

At the same time, IHC-P was used to investigate the expression and distribution of β-catenin which is considered the core molecule in the Wnt signaling pathway (Figure 6). The positive expression area of β-catenin in treated embryos was visually higher compared to the BLANK and CK groups (Figure 6A). β-Catenin was mainly expressed in the *epidermis* and placode at E13, in the inner root sheath and outer root sheath at E17, and in the epidermal collar and *epidermis* at E21 and E25 (Figure 6B). The IHC-P results supported that the promoting effects of CHIR-99021 on feather growth and feather follicle development were associated with the regulation of the Wnt signaling pathway by CHIR-99021.

### DEGs Associated With Translation, Metabolism, Energy Transport, and Stress-Related GO Terms and KEGG Pathways

**FIGURE 7:**
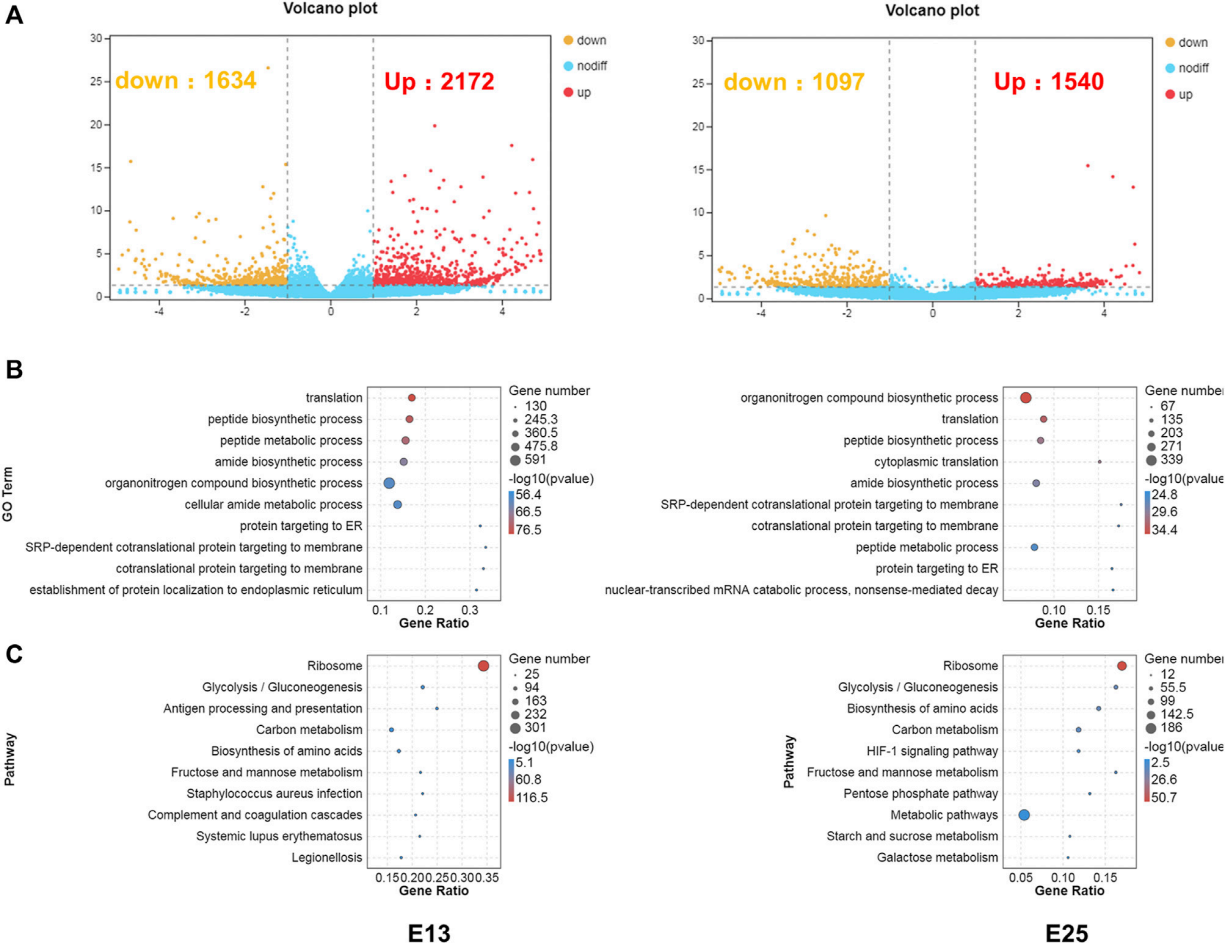
DEGs are associated with translation, metabolism, energy transport, and stress-related GO terms, and KEGG pathways. **(A)** Volcano plots of the DEGs between CK and 5,000 ng groups, respectively, at E13 and E25. **(B)** GO enrichment in biological process category of the DEGs at E13 and E25. **(C)** KEGG enrichment of the DEGs at E13 and E25. n = 3 embryos.

RNA-seq was conducted to comprehensively assess the effects of CHIR-99021 injection on transcription. A total of 3,806 and 2,637 DEGs (fold change ≥2 and *p* < 0.05) were screened between the CK and 5,000 ng groups, respectively, at E13 and E25. There were 2,172 upregulated DEGs and 1,634 downregulated DEGs at E13 and 1,540 upregulated DEGs and 1,097 downregulated DEGs at E25 (Figure 7A).

Next, GO enrichment (Figure 7B) and KEGG (Figure 7C) enrichment analyses were performed to predict the biological functions conducted by these DEGs. The top 10 enriched GO terms in the BP category at E13 were as follows: translation, peptide biosynthetic process, peptide metabolic process, amide biosynthetic process, organonitrogen compound biosynthetic process, cellular amide metabolic process, protein targeting to ER, SRP-dependent co-translational protein targeting to membrane, co-translational protein targeting to membrane, and establishment of protein localization to the endoplasmic reticulum. In addition, the top 10 enriched GO terms in the BP category at E25 were annotated in the organonitrogen compound biosynthetic process, translation, peptide biosynthetic process, cytoplasmic translation, amide biosynthetic process, SRP-dependent co-translational protein targeting the membrane, co-translational protein targeting the membrane, peptide metabolic process, protein targeting the ER, nuclear-transcribed mRNA catabolic process, and nonsense-mediated decay.

At E13, the top 10 enriched KEGG pathways were as follows: ribosome, glycolysis/gluconeogenesis, antigen processing and presentation, carbon metabolism, biosynthesis of amino acids, fructose and mannose metabolism, *Staphylococcus aureus* infection, complement and coagulation cascades, systemic lupus erythematosus, and legionellosis. The top 10 pathways at E25 with the highest representation of the DEGs were annotated in the ribosome, glycolysis/gluconeogenesis, biosynthesis of amino acids, carbon metabolism, HIF-1 signaling pathway, fructose and mannose metabolism, pentose phosphate pathway, metabolic pathways, starch and sucrose metabolism, and galactose metabolism. The detailed results of DEGs, GO enrichment, and KEGG enrichment are, respectively, listed in Supplementary Materials S2–S4.

**FIGURE 8:**
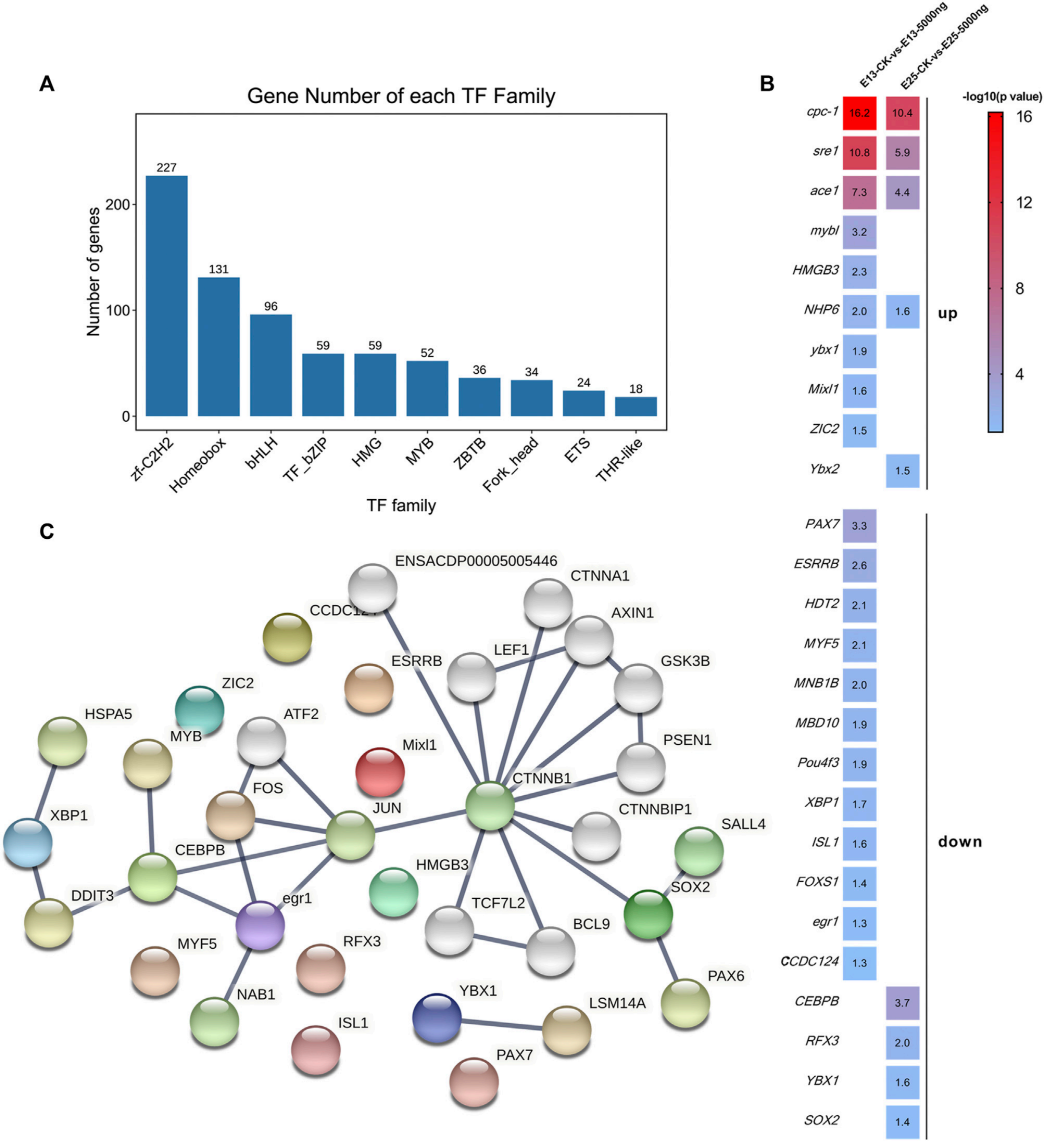
Transcription factors analyses. **(A)** Families of all transcription factors were detected in this study. **(B)** Heat map of the shared and unique differentially expressed transcription factors genes as ranked by -log10 (*p*-value). **(C)** PPI network is predicted by the differentially expressed transcription factor genes. n = 3 embryos.

The enrichment analyses illustrated that the DEGs were associated with transcription and translation. Therefore, further analyses were performed to screen transcription factors. All of the transcription factors (TFs) detected in this study were annotated in 1,055 families, and mainly in zf-C2H2, Hompbox, and bHLH families (Figure 8A). The shared and unique differentially expressed TF genes between groups at E13 and E25 were ranked by -log_10_ (*p*-value) and shown in Figure 8B. Then, the protein-protein interaction (PPI) network of these differentially expressed TF genes was constructed and predicted that CEBPB, CTNNB1, and SOX2 were the hub nodes (Figure 8C).

## Discussion

In birds, feather follicle development is one of the crucial bioprocesses during embryogenesis (Chen, et al., 2015; Ng and Li, 2018). Particularly for geese, feather follicle development to a large extent determines the feather-related economic traits (Lu, et al., 2015). Thus, understanding and regulating feather follicle development is significant to promoting the feather traits in goose farming.

Wnt signaling is very crucial for feather follicle development. The previous studies have suggested that there are a lot of DEGs in geese dorsal tissues across embryonic days and in different breeds (Liu, et al., 2020; Liu, et al., 2018; Lowe, et al., 2015; Sello, et al., 2019) and these DEGs participate in the Wnt, BMP, MAPK, and Notch pathways. With the advancement of genetic engineering techniques, such as gene knockout and gene location mutation, it has been confirmed that the Wnt signaling pathway plays a key regulatory role during feather follicle development (Andl, et al., 2002; Liu, et al., 2020; Xie, et al., 2020a). The Wnt signaling pathway and its effector β-catenin have highly dynamic regulation effects on the developmental processes of feather follicles at each stage, especially at the early feather follicle morphogenesis stage during the embryonic period. They are associated with the biological processes of the placode formation, dermal papillae function, feather follicle cycle, and proliferation, and differentiation of feather follicle stem cells (Millar, 2002; Schmidt-Ullrich and Paus, 2005; Lim and Nusse, 2013). Moreover, the activation and conduction of dermal Wnt signal paternally induce placode formation, and Wnt is the initial signal for starting up the feather follicle development program (Qiu, et al., 2020).

Small molecule inhibitors participate in the regulation of stem cell fate and the reprogramming of somatic cells (Wu, et al., 2015). CHIR-99021, as a small molecule inhibitor, can keep the Wnt signaling pathway active by inhibiting GSK-3β (Govarthanan, et al., 2020). In addition, it is also reported that CHIR-99021 has positive effects on embryonic stem cells differentiation (Ma, et al., 2019) and human diseases (Wu, et al., 2015) by modulating p53 (Wang, et al., 2015), methylation modifications, protein-coding genes expression, TF expression (Wu, et al., 2013), and long intergenic non-coding RNAs expression (Govarthanan, et al., 2020). In this study, the goose embryos were administrated with CHIR-99021 at the early feather follicle morphogenesis stage (E9) in an attempt to modulate the Wnt signaling pathway and further promote feather follicle development. The results showed that *in ovo* injection of CHIR-99021 promoted feather length, feather width, and feather follicle diameter, and feather follicle density during geese embryogenesis. However, it is notable that the promoted effects were driven by the injection dose and the time after receiving the injection. The economic value of a feather determines that a higher feather yield, feather quality, and growth rate are beneficial to the goose industry (Chen, et al., 2020a). Although strategies on nutrition and management are improving this status, some genetic and physiological bases still are limited. Initially, the feathers can be produced cyclically throughout a bird’s life beginning in the embryonic period. A certain number of feather follicles have been developed during the embryonic period (Jenni, et al., 2020; Prum, 1999). Moreover, feather quantity and quality were determined by feather follicle density and diameter to a large extent (Chen, et al., 2012; Jenni, et al., 2020). Thus, modulation of feather follicle development during embryogenesis has profound and lasting implications for feather traits. Our data showed that *in ovo* injection of CHIR-99021 is a potential strategy to promote feather follicle development and feather quality and quantity.

Studies have confirmed that hair and/or feather follicle development can be promoted by activating the Wnt/β-catenin signaling pathway (Gentile and Garcovich, 2019; Qiu, et al., 2017; Zhou, et al., 2018). In previous studies, it was shown that *in ovo* injection of methionine improved feather follicle development in chick embryos by activating the Wnt/β-catenin signaling pathway (Chen, et al., 2020b). In contrast, *in ovo* injection of Dickkopf-1 (a target Wnt/β-catenin specific inhibitor) in chick embryos reduced feather follicle development and feather quality during both embryonic and post-hatching periods by inhibiting β-catenin, TCF4, Cyclin D1, and c-Myc (Xie, et al., 2020b). In mammals, CHIR-99021 stimulated human dermal papilla spheroids, which helped in the formation of hair follicles and the production of reconstituted hair follicle-enriched human skin (Sun, et al., 2021; Yoshida, et al., 2019). The molecular mechanisms are considered that CHIR-99021 inhibits GSK-3β to prevent β-catenin degradation by the ubiquitin-proteasome system, which results in nuclear translocation of β-catenin to promote the upstream transmembrane protein FZD4 expression. In addition, β-catenin quickly combines with the transcription factors LEF1 and TCF4 in the nucleus to promote the target c-Myc expression further to stimulate the dermal cell condensation and the proliferation and migration of feather follicle stem cells niched in the outer root sheath (Wu, et al., 2013). In our study, the western blotting results also supported that CHIR-99021 can upregulate Wnt signals consisting of FZD4, β-catenin, LEF1, TCF4, and c-Myc by inhibiting GSK-3β. The IHC-P results showed that the expression level of β-catenin was increased in the embryos that received CHIR-99021. The distribution of β-catenin mainly was in the epidermis and placode at E13 and in root sheath at E17, where dermal cell condensation for initial feather follicle morphogenesis occurs. Although, the distribution of β-catenin at E21 and E25 mainly was in the epidermis and epidermal collar. The epidermis contains a large number of feather follicle stem cells with multipotent differentiation ability and participation in the formations of the epidermis and sebaceous glands. Therefore, our data provide evidence that, at the protein level, *in ovo* injection of CHIR-99021 promoted feather follicle development in goose embryos by modulating the Wnt signaling pathway.

However, recent studies have pointed out that CHIR-99021 also drives transcription (Delepine, et al., 2021; Wang, et al., 2015). We thereby conducted RNA-seq to comprehensively understand the mechanisms of CHIR-99021 in promoting feather follicle development in goose embryos. The DEGs number at E13 was less than that at E25 and a lot of unique and shared DEGs were observed between E13 and E25, indicating the effect of *in ovo* injection of CHIR-99021 changed over time after receiving the injection. The top DEGs between groups at E13 and E25 were *Myh4*, *MYLPF*, and *MYBPH* (subunits of *MYH* and *MYL* families) (Ayuso, et al., 2015; Ghosh, et al., 2016; Xu, et al., 2013), *pvalb* and *pvalb2* (*parvalbumin* genes) (Chu, et al., 2015; da Silva-Gomes, et al., 2019; Zhang, et al., 2017), *CKM* (Gronek, et al., 2013) and *atp2a1* (Jin, et al., 2014; MacLennan, et al., 1985), are associated with development by regulating the muscle growth and energy transfer (Ayuso, et al., 2016; da Silva-Gomes, et al., 2019; Xue, et al., 2017). Therefore, we speculate that the effects of CHIR-99021 on promoting feather follicle development might be associated with the expression of muscle growth and energy transfer-related genes.

GO and KEGG enrichment analyses of the DEGs between groups showed that there were shared enriched GO terms and KEGG pathways between E13 and E25. Most of the top 20 enriched GO terms in the biological process category and KEGG pathways are associated with transcription, translation, and protein synthesis, and energy metabolism. Notably, the first enriched KEGG pathway was ribosomes at both E13 and E25. Similar results were found in a study on the sheep hair follicle; however, Lv et al. (2020) reported that the nutrition-driven hair follicle development is associated with the enriched ribosome pathway, which suggests that hair/feather follicle development can be affected by the ribosome pathway. During the embryonic period in birds, rapid morphogenesis, differentiation, and development of various organs require support from cell division, consisting of quick connection of gene expression programs, acceleration of the translation programs, and quick regulation of protein synthesis (Guo, 2018). During the process, the control of translation is considered the main determining factor of the protein level in the cell, while the ribosome, as the translation machine in protein synthesis plays a central role in the regulation of gene expression (Bowman, et al., 2020; Javed and Orlova, 2019; Natchiar, et al., 2015; Poitevin, et al., 2020). Ribosome and translational control are directly linked with stem cell homeostasis and control of cell fate and play vital roles in the translational regulatory network that controls the identity of stem cells (Gabut, et al., 2020). Therefore, the biological functional accomplishment of CHIR-99021 possibly involves maintaining the high-speed operation of ribosomes, remodeling and priming gene expression toward differentiation programs, regulating translation, and accelerating protein synthesis in feather follicle stem cells.

The biosynthesis of ribosomes is a complicated process. Metabolism and energy are essential to support the process and development depends on continuous metabolism. However, the metabolites provide accumulation, energy, and reducing power for growth, development, and reproduction (Haeusler, et al., 2014). The metabolic pathways take place in the cytoplasm and mitochondria of cells by glucose or fatty acids to provide most of the cellular energy for animals (Judge, et al., 2020). Feather follicle stem cells acquire energy by participating in aerobic glycolysis, lactic acid production, and the pentose phosphate pathway (PPP), to maintain their proliferation (Badolati, et al., 2018; Flores, et al., 2017; Williams, et al., 1993). In our study, many metabolism-related pathways were enriched and might provide the required energy to support the biological processes of transcription and translation induced by CHIR-99021.

We noticed that the glycolysis/gluconeogenesis pathway was the second enriched pathway at both E13 and E25. The glycolysis/gluconeogenesis pathway has been reported in hair follicle studies (Guo, et al., 2020; Kim, et al., 2020). During hair follicle development in sheep fetuses, screened differentially abundant proteins were associated for metabolism and skin development and were enriched to the glycolysis/gluconeogenesis pathway. This suggests that this pathway may be one of the hair follicle development-related pathways (Guo, et al., 2020). Moreover, Kim, et al. (2020) also found that a higher level of glycolysis provides energy for hair follicle development in hair follicle stem cells. We also noticed that the PPP was enriched in this study. PPP plays a fundamental component in the cellular metabolism. PPP is a branch of glycolysis that is the key step of glucose metabolism, which is necessary for the synthesis of ribonucleotides and is also the main source of nicotinamide adenine dinucleotide phosphate (NADPH) (Patra and Hay, 2014; Stincone, et al., 2015). Therefore, metabolism-related pathways provide energy to adapt the accelerated biosynthesis of ribosomes further to coordinate feather follicle development. We suggest that the metabolism-related pathways might play an adapting role in feather follicle development.

In the present study, stress-related pathways, such as the complement and coagulation cascades, and HIF-1 signaling pathways, were also enriched. Phylogeny considers that genes in the complement and coagulation systems existed before vertebrates appeared (Dzik, 2019). Complement and coagulation may prevent the overactivation and tissue damage during rapid feather follicle development (Ji, et al., 2018). HIF-1 is the main regulator of oxygen homeostasis (Semenza, 2010), and regulates various adaptive physiological responses to hypoxia via promoting O_2_ transport (Semenza, 2014; Yoon, et al., 2006), and also can activate the transcription of genes encoding glucose transporters and glycolytic enzymes (Semenza, 2010; Semenza, 2014). Thus, this study also suggests that embryos have ability to balance the rapid development-induced stress by mobilizing the anti-stress system.

It is confusing that Wnt proteins had significant differences at the protein level as indicated in western blotting results, while the Wnt signaling pathway was not significantly enriched as shown in RNA-seq results. On the one hand, the levels between mRNA and protein are not completely positive in correlation. On the other hand, CHIR-99021 may act in the stages of transcription and post-transcription. Therefore, we speculate that CHIR-99021 might independently and/or mutually affect feather follicle development at the gene and protein levels.

A comprehensive understanding of the complex changes in gene expression requires a global analysis of the interactions between epigenetic, transcription, and translation mechanisms. We performed TF analyses, and found downregulated *CEBPB* at E25 and upregulated *sre1* at E13 and E25. *CEBPB* is associated with poultry growth and is considered an inhibitor of myogenesis. Xue, et al. (2017) also reported that higher expression of *CEBPB* caused lower muscle growth in chickens, while *sre1* was upregulated during the stretch overload of the chick back muscles (Carson and Booth, 1998; Carson, et al., 1996). We also noticed that some differently expressed TF, such as *NHP6* (Kurat, et al., 2017), *Mixl1* (Chapman, et al., 2013; Hart, et al., 2002), and *PAX7* (Buckingham and Relaix, 2015; Soglia, et al., 2020), are, respectively, associated with chromatin transcription, embryogenesis, and protein modification. In addition, the current study has reported that the *PAX7* gene is related to the growth of poultry (Soglia, et al., 2020), and maybe a candidate gene for goose growth traits and marker-assisted selection (Wang, et al., 2017). The PPI network of these differently expressed TF genes predicts that some hub molecules (CTNNB1, LEF1, and GSK-3β) were associated with the TF, and predicts that *SOX2* is one of the key nodes (Novak, et al., 2020). The mesenchymal cells in dermal condensates express *SOX2* during the formation of the dermal sheath and the formation of dermal papilla (Driskell, et al., 2009; Iida, et al., 2020; Rendl, et al., 2006). *SOX2* can induce an localized growth zone laying, the foundation for the formation of hair follicles (Wu, et al., 2018). Therefore, we suggest CHIR-99021 possibly by modulating TF to adapt to the rapid muscle development surrounding feather follicles and the changed transcription.

However, some limitations of the study should be noted. This study used *de novo* transcriptome to reveal the transcriptional changes in embryos treated with CHIR-99021. Geese are not common model animals and have limited genomic information. The extension of related genomic information in geese needs further elucidation.

## Conclusion

In conclusion, goose embryos *in ovo* injection of 5,000 ng CHIR-99021 at E9 (dermal cell condensation stage) promoted feather length, feather width, feather follicle diameter, and feather follicle density. Goose embryos *in ovo* injection of 5,000 ng CHIR-99021 at E9 activated the Wnt signaling pathway by inhibiting GSK-3β at the protein level. Moreover, *de novo* transcriptome analyses suggest that the translation, metabolism, energy transport, and stress-related genes, TF, and pathways were enriched possibly to adapt and coordinate the promoted effects of CHIR-99021 on feather follicle development. This study suggests that *in ovo* injection of CHIR-99021 is a potential strategy to improve feather follicle development and feather-related traits for goose farming and reveals profiling of the Wnt signaling pathway and transcriptome in dorsal tissues of goose embryos for further understanding feather follicle development and provides a theoretical basis for understanding the mechanism of CHIR-99021 in promoting feather follicle development.

## Data Availability

The datasets presented in this study can be found in online repositories. The name of the repository and accession number can be found in the following: National Center for Biotechnology Information (NCBI) BioProject, https://www.ncbi.nlm.nih.gov/bioproject/, PRJNA792024. The other original contributions presented in the study are included in the article/Supplementary Materials, further inquiries can be directed to the corresponding authors.

## Abbreviations

GSK-3β, glycogen synthase kinase 3β; NS, normal saline; DEGs, differently expressed genes; PPP, production and pentose phosphate pathway; and NADPH, nicotinamide adenine dinucleotide phosphate.
