# Supplementary material for: In Ovo Injection of CHIR-99021 Promotes Feather Follicle Development via Modulating the Wnt Signaling Pathway and Transcriptome in Goose Embryos (Anser cygnoides)
Source: Front Physiol. 2022 May 20;13:858274. doi: 10.3389/fphys.2022.858274 (PMC9164139; doi:10.3389/fphys.2022.858274)
Supplement: Supplementary file 1 [file DataSheet1.DOCX]

Table S1. Quality control of RNAs for sequencing.

| Sample ID | Concentration (ng/μl) | RIN |
| --- | --- | --- |
| E13-CK-1 | 633 | 10 |
| E13-CK-2 | 642 | 10 |
| E13-CK-3 | 484 | 10 |
| E25-CK-1 | 205 | 7.7 |
| E25-CK-2 | 212 | 7.7 |
| E25-CK-3 | 317 | 7.9 |
| E13-5000ng-1 | 633 | 10 |
| E13-5000ng-2 | 660 | 10 |
| E13-5000ng-3 | 846 | 10 |
| E25-5000ng-1 | 308 | 7.5 |
| E25-5000ng-2 | 338 | 7.8 |
| E25-5000ng-3 | 211 | 7.3 |

Table S2. Quality control of RNA-seq data.

| Sample | Raw reads | Clean reads (%) | Clean bases (bp) | Q20 (%) | Q30 (%) | GC (%) |
| --- | --- | --- | --- | --- | --- | --- |
| E13-CK-1 | 43172664 | 42984474 (99.56%) | 6419397244 | 6318987962 (98.44%) | 6130436724 (95.50%) | 3561911177 (55.49%) |
| E13-CK-2 | 47502652 | 47290466 (99.55%) | 7064922182 | 6951637082 (98.40%) | 6741078763 (95.42%) | 3912895209 (55.38%) |
| E13-CK-3 | 49613634 | 49368992 (99.51%) | 7375498468 | 7257150541 (98.40%) | 7040144386 (95.45%) | 4129073298 (55.98%) |
| E13-5000ng-1 | 40461122 | 40265148 (99.52%) | 6014736113 | 5921629918 (98.45%) | 5750349743 (95.60%) | 3346399211 (55.64%) |
| E13-5000ng-2 | 43569940 | 43359136 (99.52%) | 6476931210 | 6378157760 (98.47%) | 6196608480 (95.67%) | 3610489069 (55.74%) |
| E13-5000ng-3 | 43285838 | 43066234 (99.49%) | 6425698711 | 6323117408 (98.40%) | 6135476388 (95.48%) | 3596751404 (55.97%) |
| E25-CK-1 | 41452662 | 41268504 (99.56%) | 6159788147 | 6060637972 (98.39%) | 5882368282 (95.50%) | 3388415625 (55.01%) |
| E25-CK-2 | 51526280 | 51301104 (99.56%) | 7665374732 | 7539933548 (98.36%) | 7326400173 (95.58%) | 4319224594 (56.35%) |
| E25-CK-3 | 44077534 | 43875188 (99.54%) | 6559579288 | 6452769417 (98.37%) | 6268026322 (95.56%) | 3679154489 (56.09%) |
| E25-5000ng-1 | 45032578 | 44832318 (99.56%) | 6695206393 | 6580364883 (98.28%) | 6369038827 (95.13%) | 3576356695 (53.42%) |
| E25-5000ng-2 | 46867052 | 46646516 (99.53%) | 6965044674 | 6847703017 (98.32%) | 6644038466 (95.39%) | 3969008512 (56.98%) |
| E25-5000ng-3 | 41940750 | 41745374 (99.53%) | 6235063265 | 6131044537 (98.33%) | 5945937176 (95.36%) | 3456474857 (55.44%) |

Table S3. Primers for qPCR.

| Gene | Primer sequences (5’-3’) | Product size (bp) | Annealing Tm (̊C) | GC% | Accession numbers |
| --- | --- | --- | --- | --- | --- |
| *FZD4* | AGCCTGGAGAAGAGTGCCACAG  TCCTTCGCTGACCTGCTGTAGAG | 124 | 64.23  63.85 | 59.09  56.52 | XM_013199825.1 |
| *CTNNB1* | CCAGGTTGGTGGCATTG  TGGTGAGGTGACGGAGC | 101 | 54.4  58.0 | 58.8  64.7 | XM_013184751.1 |
| *RPL36* | TAGTGCTTGTGTTCGTTGCGA  ATAGGGTGCAAAGCCACAGA | 112 | 60.80  59.30 | 47.62  50.00 | XM_013196259.1 |
| *SPARC* | AAAGCCCGCAGATTCCTC  TCATCTTTCAGCAAACCCTCG | 87 | 57.35  58.57 | 55.56  47.62 | XM_013175993.1 |
| *c-Myc* | TGTTTGCAGTGTTGGTAGCC  ATGCTTCAGAAAGTCCAGTGC | 71 | 58.97  58.84 | 50.00  47.62 | XM_013184287.1 |
| *β-actin* | GCGGCATGCCACACCGTGCCCATCTATGAG  GCGAAGCTTGGCCATCTCCTGCTCGAAGT | 205 | 71.8  68.7 | 63.3  58.6 | NM_205518.1 |
| *WNT11* | AGAGCTGCGAGACATTGCAT  GTCTTGTTGCATTGCCTGTCC | 22 | 60.11  60.34 | 50.00  52.38 | XM_013191254.1 |
| *BKJ* | GCAAAGTCCTACCAAGCGGG  TTCACAGACGAGAGCAAGGC | 296 | 61.31  60.32 | 60.00  55.00 | NM_204846.1 |
| *BMP4* | TTCCACCATGAAGAGCACCTG  AGGTTGAAGACGAAGCGGAT | 74 | 60.27  59.39 | 52.38  50.00 | NM_205237.3 |
| *GAPDH* | CTGGCATTGCACTGAACGAC  CATCAAGTCCACCACACGGT | 89 | 60.11  60.25 | 55.00  55.00 | XM_013199522.1 |
| *MAPK1* | AGCTTCTGGTTCTGCCTACG  CATGCCTGAAGCGCAGTAAG | 131 | 59.75  59.62 | 55.00  55.00 | XM_013173549.1 |
| *DKK1* | CGGAACTCGGCACTGACC  GGTTTGCAGATCTTGGACCAG | 118 | 60.43  59.18 | 66.67  52.38 | XM_040702954.1 |
| *FOXO3* | CATCACGAAGTCTGGGGCTT  TATTTGCTCGGCACCTCTGG | 146 | 60.04  60.11 | 55.00  55.00 | XM_013175636.1 |
| *NDRG1* | GGAAGAGGCTATCACTCGCC  CACATGGTGACATGGACCGA | 90 | 59.97  60.04 | 60.00  55.00 | XM_013192663.1 |
| *RRAS2* | GGCACGTGCAAGTCCTATT  TGTTCACGCATGGCTCCAAA | 146 | 58.15  60.82 | 52.63  50.00 | XM_013189079.1 |
| *SRF* | TGGTCAACTTGGACACCTCG  GTACGAGAACGAATGGCTGC | 119 | 59.89  59.35 | 55.00  55.00 | XM_013194954.1 |
| *PTCH1* | GGAGTCTTGCTGGTTGCTCT  ACTTGCTCCTGTTCGCTTCA | 225 | 59.96  59.89 | 55.00  50.00 | XM_013198795.1 |


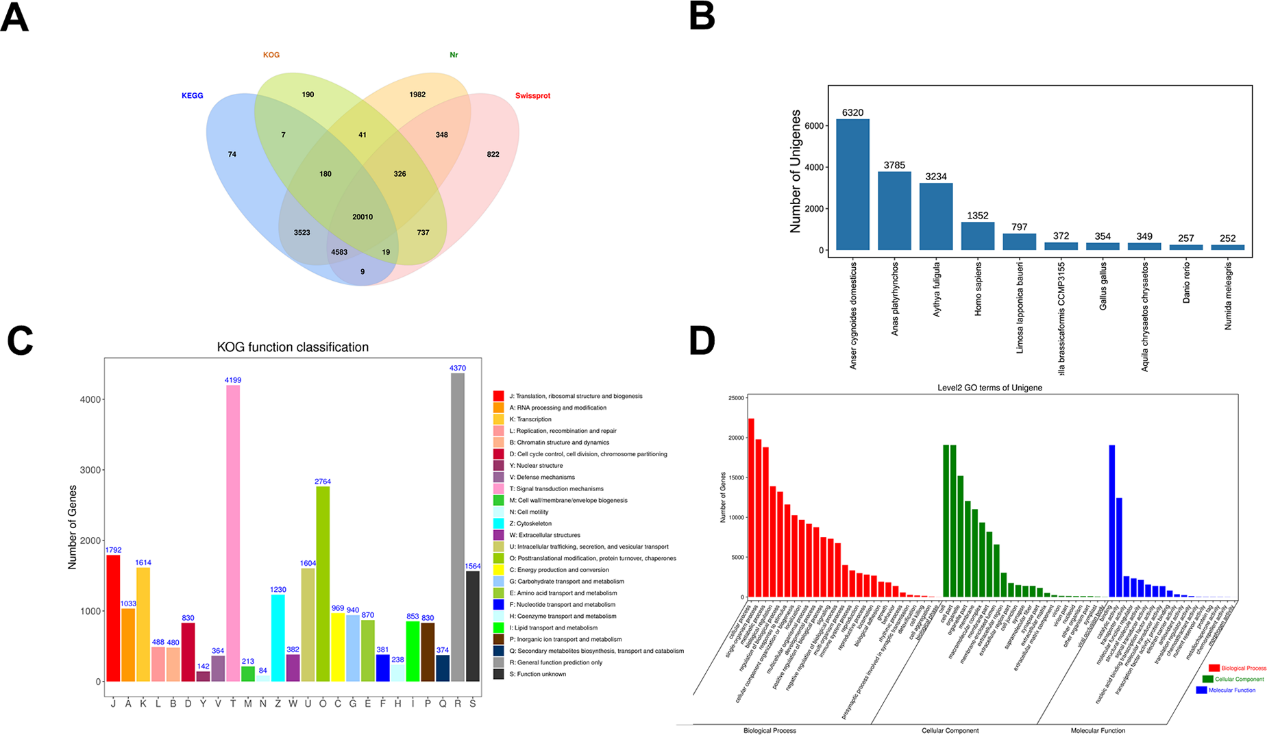


Fig. S1. Unigene annotation. (A) Annotation summary of Nr, SiwssProt, KEGG and COK/KOG databases. (B) The number of homologous sequences between species. The unigenes’ function annotations in COK/KOG (C) and GO databases (D).


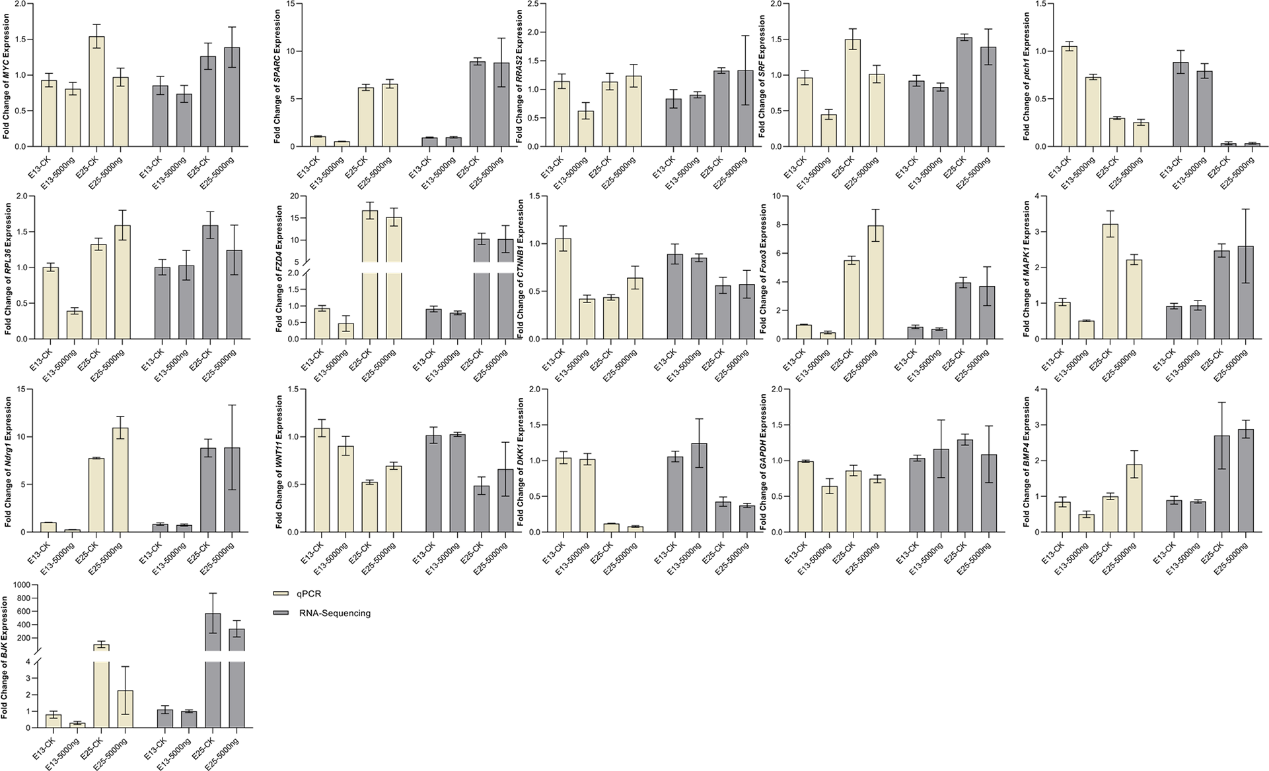


Fig. S2. Validation of *de novo* transcriptome by qPCR.
